# Hospital Appeals in Time of War

**Published:** 1914-09-26

**Authors:** 


*To the Editor of* The Hospital.

### Hospital Appeals in Time of War.

*To the Editor of* The Hospital.

Sir,—Abandoning the stereotyped phrase, "a renewal of your support," in my letters when appealing for the continuance of annual subscriptions, I substituted instead an appeal for subscribers of the hospital to rally round it during this dark period, pointing out that in the near future there were likely to be many more claims upon the hospital, which would tax its resources to the utmost.

The first answer received was most encouraging, and ran as follows: "I fully appreciate your responsibilities just now, and therefore, instead of the ordinary subscription (one guinea), I have pleasure in sending you a special donation of ten guineas." It remains to be seen whether this will prove to be an isolated case or not.—Yours faithfully,

Provincial Hospital Secretary.

[In raising this question in our Notes last week we referred to the tactics of an institutional secretary in Glasgow, who suggested asking for a half or quarter of the customary subscription instead of "a renewal of your support." We invited suggestions, and shall be glad to publish further letters on the proper policy to pursue. Another example is given in our Notes (page 688).—Ed. The Hospital.]

